# Genetic differentiation among *Aedes aegypti* populations from different eco-geographical zones of India

**DOI:** 10.1371/journal.pntd.0011486

**Published:** 2023-07-27

**Authors:** Melveettil Kishor Sumitha, Mariapillai Kalimuthu, Mayandi Senthil Kumar, Rajaiah Paramasivan, Narendran Pradeep Kumar, Ittoop Pulikkottil Sunish, Thiruppathi Balaji, Devojit Kumar Sarma, Devendra Kumar, Devi Shankar Suman, Hemlata Srivastava, Ipsita Pal Bhowmick, Keshav Vaishnav, Om P. Singh, Prabhakargouda B. Patil, Suchi Tyagi, Suman S. Mohanty, Tapan Kumar Barik, Sreehari Uragayala, Ashwani Kumar, Bhavna Gupta

**Affiliations:** 1 ICMR-Vector Control Research Centre, Field Station, Madurai, India; 2 ICMR-Vector Control Research Centre, Field Station, Kottayam, India; 3 ICMR-Regional Medical Research Centre, Port Blair, Andaman & Nicobar Islands, India; 4 ICMR-National Institute for Research in Environmental Health, Bhopal, India; 5 Department of Zoology, Mohanlal Sukhadia University, Udaipur, Rajasthan, India; 6 Estuarine Biology Regional Centre, Zoological Survey of India, Gopalpur-on-Sea, Ganjam, Odisha, India; 7 School of Biological Sciences, Institute of Management Studies (University Courses Campus), Ghaziabad, Delhi, India; 8 ICMR-Regional Medical Research Centre, North East, Dibrugarh, India; 9 Surat Municipal Corporation, Surat, India; 10 ICMR-National Institute of Malaria Research, New Delhi, India; 11 Maharashtra Hybrid Seeds Company Private Limited, Jalna, Maharashtra, India; 12 ICMR-National Institute for Implementation Research on Non-Communicable Diseases, Jodhpur, India; 13 Medical Entomology Laboratory, Post Graduate Department of Zoology, Berhampur University, Ganjam, Odisha, India; 14 ICMR-National Institute of Malaria Research, Field Unit, Bengaluru, India; 15 ICMR-Vector Control Research Centre, Puducherry, India; International Centre for Genetic Engineering and Biotechnology, INDIA

## Abstract

The present study explicitly evaluated the genetic structure of *Aedes aegypti* Linn, the vector of dengue, chikungunya, and Zika viruses, across different geo-climatic zones of India and also elucidated the impact of ecological and topographic factors. After data quality checks and removal of samples with excess null alleles, the final analysis was performed on 589 individual samples using 10 microsatellite markers. Overall findings of this study suggested that, *Ae*. *aegypti* populations are highly diverse with moderate genetic differentiation between them. Around half of the populations (13 out of 22) formed two genetic clusters roughly associated with geographical regions. The remaining nine populations shared genetic ancestries with either one or both of the clusters. A significant relationship between genetic and geographic distance was observed, indicating isolation by distance. However, spatial autocorrelation analysis predicted the signs of long-distance admixture. Post-hoc environmental association analysis showed that 52.7% of genetic variations were explained by a combination of climatic and topographic factors, with latitude and temperature being the best predictors. This study indicated that though overall genetic differentiation among *Ae*. *aegypti* populations across India is moderate (*F*_*st*_ = 0.099), the differences between the populations are developing due to the factors associated with geographic locations. This study improves the understanding of the *Ae*. *aegypti* population structure in India that may assist in predicting mosquito movements across the geo-climatic zones, enabling effective control strategies and assessing the risk of disease transmission.

## Author summary

*Aedes aegypti* is a mosquito species that transmits several arboviruses including dengue, chikungunya, Zika, etc. This species was originally found in Africa but is now extensive in all the continents except Antarctica. The spread of this species is of concern as the movement of infected mosquitoes can spread the viruses from one place to another. Anthropogenic changes such as climate warming and increasing urbanization are boosting the spread. This study focuses on analyzing the genetic structure of *Ae*. *aegypti* in India and how it is affected by the local topological and climatic factors. We analyzed *Ae*. *aegypti* populations from different geographical regions in India covering the parts from North to South and East to West and analyzed them using a panel of microsatellite markers. Our analysis suggested that *Ae*. *aegypti* populations in India are genetically closer to each other irrespective of geographical distance between them which indicates high gene flow among the populations. However, signs of genetic structure were also observed owing to the factors associated with geographical locations. This research provides a broader picture of the nature and dynamics of the mosquito populations among eco-geographical regions across a larger area of the country, which would provide a valuable reference base for genetic studies and for novel mosquito control strategies.

## Introduction

One of the fundamental challenges in population genetics is to understand the pattern of genetic structure and delineate the factors shaping it. Genetic differentiation is determined by the amount of gene flow (migration) across populations. The migration of mosquitoes across space is generally influenced by their biological and ecological behavior, population history, eco-geographic barriers, and other local environmental factors [1–5]. A clear understanding of these issues is important in several contexts of disease control including; the movement of vectors that can disseminate pathogens, insecticide resistance genes/mutations, and released mosquito populations carrying sterility factors or other transgenic genes for controlling disease transmission [6–10].

*Aedes aegypti* is one of the most important mosquito species worldwide as it is the major vector of dengue, chikungunya, Zika, and other arboviruses and has contributed to several disease outbreaks worldwide. It is a human commensal that not only feeds on humans [11,12] but can complete its life cycle within human dwellings. The flight range of the mosquito species is further restricted to few hundred meters if sufficient breeding and feeding options are available nearby [13–17]. However, human movement (infrastructure, transport, connectivity, trade, etc.) has a significant role in determining the genetic structure of this species [18–21]. For example, *Ae*. *aegypti* populations from urban areas with good connectivity are less differentiated than the populations from areas with restricted human movements [22–27]. Thus, the genetic structure and the underlying factors may vary among geographical regions.

Genetic structure of *Ae*. *aegypti* has been studied worldwide [23]. The most extensive analysis to date genotyped 3632 mosquito samples from 79 collections across the globe with 12 microsatellite loci [23]. This analysis identified two major genetic clusters at the global level; ancestral populations from Africa and that from other parts of the world [23]. The populations outside Africa were found highly diverse and the diversity was associated with the geography and migration history of *Ae*. *aegypti*. Genetic distinctness and limited gene flow among populations are crucial factors in mosquito control and thus understanding the local vector population dynamics is important for maximum benefits.

In India, *Ae*. *aegypti* is most prevalent in urban areas [28–31], but over the years the mosquito species have also been reported to breed in rural areas [31–33]. *Aedes* control mainly relies on source reduction, environmental manipulation, and sanitation [34,35]. Notably, laboratory studies on modern vector control technologies are also making their way for the future [36–38]. However, our knowledge of the genetic structure of this mosquito species in India is scarce. The geographical vastness of the country and the huge climatic and ecological variations has a tremendous effect on mosquito populations. For example, a recent study demonstrated significant differences in the survival and reproductive strategies of *Ae*. *aegypti* and *Aedes albopictus* from desert and coastal regions of India [39,40]. *Ae*. *aegypti* was found better suited to desert conditions while *Ae*. *albopictus* was more adapted to coastal environment of Kolkata. Understanding the population dynamics of the mosquito species from different eco-geographical regions is important. The genetic studies on *Ae*. *aegypti* populations in India available so far are geographically localized to a particular city or state [22,33,41–43] or have used a few genetic markers [33,41]. There has been no genetic study on *Ae*. *aegypti* at the national level. With this in mind, the present study was carried out to explore the genetic structure of the species across the country and the possible factors that determine the spatial structuring among them.

## Material methods

### Mosquito sampling

**Fig 1:**
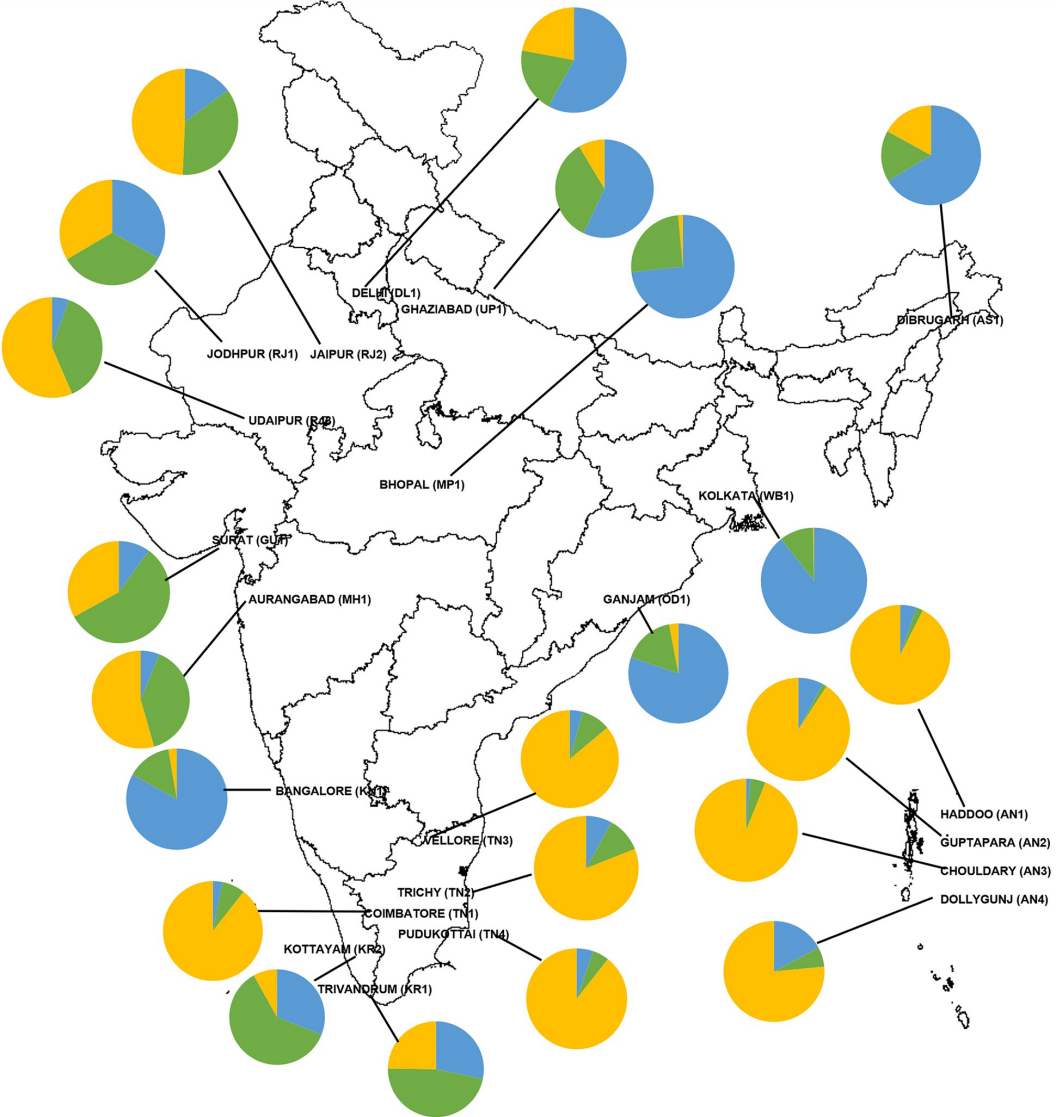
Location of *Aedes aegypti* populations sampled. Pie charts indicate the proportion of genetic ancestry assigned to individuals of each population by the Bayesian clustering method. Colors in the pie chart indicate the genetic ancestries. The state boundary shape file was obtained from the survey of India (https://onlinemaps.surveyofindia.gov.in/FreeOtherMaps.aspx) and the map was created using DIVA-GIS (https://www.diva-gis.org/).

*Aedes aegypti* mosquitoes were collected from 22 different locations across India, from 2018 to 2021. The sampling locations and their characteristics are given in Table 1 and Fig 1. The clustering of sampling sites based on 19 bioclimatic variables is shown in S1 Fig. The data on bioclimatic variables were retrieved from the WorldClim database using the principal coordinates for each sampling site (S1 Table). The nineteen bioclimatic variables included monthly, quarterly and yearly data on average and extreme values of temperature and rainfall (S1 Table). The geographical distance between the sampling sites ranged from ~6 km to ~2800 km and within each collection site, sampling was done from several locations covering larger areas of the city. Adults were collected using aspirators or Prokopacks. However, the majority of the samples were collected through larval pipetting. Collected larvae/pupae were reared in the laboratory upto adulthood and then species were identified using microscopes. If the samples were from larvae/pupae, only one mosquito per container was selected for further analysis to reduce the chances of analyzing siblings. Adult *Ae*. *aegypti* mosquitoes were stored in 95% ethanol for further processing. A minimum of 30 samples per site were randomly selected for genetic analysis, but all samples were used for the sites where less than 30 mosquitoes were collected.

**Table 1:** Geographical characteristics and genetic diversity estimate of *Ae*. *aegypti* populations collected from different geographical regions of India.

Sl. No.	Sampling locations	State/UT	Code	Latitude	Longitude	Altitude (msl)	N	*Na*	*Ne*	*H*_*o*_	*H*_*e*_	*F*_*IS*_
1	HADDOO	Port Blair (Andaman & Nicobar) (AN)	AN1	11°40’23”	92°43’15”	16	30	4.600	2.480	0.856	0.581	**-0.474**
2	GUPTAPARA		AN2	11°33’45”	92°39’37”	19	29	4.400	2.808	0.837	0.590	**-0.429**
3	CHOULDARY		AN3	11°38’25”	92°39’45”	20	17	3.900	2.212	0.579	0.493	-0.166
4	DOLLYGUNJ		AN4	11°38’07”	92°42’40”	11	29	5.300	2.720	0.728	0.598	**-0.218**
5	JODHPUR	Rajasthan (RJ)	RJ1	26°14’29”	73°01’26”	223	28	4.600	2.393	0.564	0.552	-0.021
6	JAIPUR		RJ2	26°55’01”	75°47’10”	435	30	4.600	2.331	0.587	0.509	**-0.145**
7	UDAIPUR		RJ3	24°35’10”	73°42’44”	570	29	4.600	2.493	0.530	0.539	-0.007
8	TRIVANDRUM	Kerala (KR)	KR1	8°31’36”	76°56’09”	52	28	6.000	2.380	0.652	0.552	-0.171
9	KOTTAYAM		KR2	9°35’37”	76°31’18”	8	28	5.100	2.335	0.550	0.517	-0.057
10	SURAT	Gujarat (GU)	GU1	21°10’23”	72°49’50”	27	25	4.400	2.237	0.482	0.520	**0.069**
11	GHAZIABAD	Haryana (HR)	HR1	28°40’18”	77°27’12”	223	26	5.500	2.366	0.473	0.511	**0.075**
12	KOLKATA	West Bengal (WB)	WB1	22°34’49”	88°21’46”	80	24	2.300	1.804	0.205	0.354	**0.520**
13	DIBRUGARH	Assam (AS)	AS1	27°28’26”	94°54’42”	108	24	3.700	2.093	0.336	0.420	**0.234**
14	BHOPAL	Madhya Pradesh (MP)	MP1	23°15’45”	77°24’41”	495	24	5.800	2.693	0.562	0.584	**0.047**
15	BANGALORE	Karnataka (KN)	KN1	12°58’38”	77°35’37”	900	32	4.800	2.461	0.432	0.514	**0.202**
16	BEHRAMPUR	Odisha (OD)	OD1	19°23’15”	85°03’02”	25	32	4.900	2.457	0.464	0.511	**0.116**
17	DELHI	Delhi (DL)	DL1	28°42’43”	77°06’02”	192	24	5.100	2.409	0.440	0.542	**0.162**
18	AURANGABAD	Maharashtra (MH)	MH1	19°52’43”	75°20’35”	596	26	4.300	2.497	0.416	0.506	**0.094**
19	COIMBATORE	Tamil Nadu (TN)	TN1	11°01’10”	76°57’19”	446	30	5.600	2.734	0.602	0.551	-0.088
20	TRICHY		TN2	10°47’33”	78°42’15”	73	29	5.400	2.838	0.645	0.564	-0.145
21	VELLORE		TN3	12°55’08”	79°07’57”	230	29	5.800	2.837	0.632	0.565	-0.126
22	PUDUKOTTAI		TN4	10°22’58”	78°48’03”	91	16	3.900	2.662	0.601	0.521	-0.166
	Mean						26.77	4.755	2.465	0.553	0.527	-0.032

*N*: sample size; *N*a: number of alleles per locus, *H*_o_: observed heterozygosity; *H*_e_: expected heterozygosity; *F*_*IS*:_ inbreeding coefficient; significant *F*_*IS*_ values shown in bold

### Genotyping of adult mosquito samples

Genomic DNA was isolated from individual mosquitoes using DNeasy Blood & Tissue Kit (Qiagen, USA) as per the manufacturer’s protocol. Genotyping was performed using 12 microsatellite markers, including four tri-nucleotide repeats (A1, B2, B3, A9) and eight di-nucleotide repeats (AC2, CT2, AG2, AC4, AC1, AC5, AG1, and AG4) [24,44]. Amplification was performed with Qiagen Type-it-microsatellite kit using fluorescently labeled primers as described earlier [22]. Each forward primer had a M13 tail and two M13 primers each tagged with FAM and HEX, respectively were used to obtain fluorescent labeled amplified products. The labeling of the primers was categorized based on the size of the product as described in Brown *et al*., (2011) such as A9, AC4, AC1, AC5, A1, AC2 were tagged with FAM and B2, B3, AG1, AG5, CT2, AG2 with HEX [24]. Amplifications were checked in 2% agarose gel and the fragment analysis was done with ABI DNA analyzer 3730XL at Rajiv Gandhi Centre for Biotechnology (RGCB, Thiruvananthapuram). The genotyping analysis was done using Geneious Prime (Biomatters, NZ).

### Genetic diversity parameters

Standard genetic parameters such as the average number of alleles (*Na*), the number of effective alleles (*Ne*), expected and observed heterozygosity (*H*_*e*_ and *H*_*o*_, respectively), and inbreeding coefficient (*F*_*IS*_) were estimated with GenAlEx 6.5 [45]. Microchecker 2.2.3 was used to estimate the null allele frequency for each marker [46]. To establish the impact of null alleles, global *F*_*st*_ as well as locus-wise *F*_*st*_ was estimated with and without null alleles using FreeNA [47]. The statistical significance of the difference between the *F*_*st*_ estimates was tested using t-test with p-value < 0.05. The linkage disequilibrium among the locus-pairs and Hardy Weinberg Equilibrium (HWE) for each location was tested by applying Fisher’s exact test in Genepop 3.4 [47]. The statistical significance of the estimates was adjusted with Bonferroni corrections [48,49].

### Genetic differentiation and population clustering

Several methods were applied to determine the clustering pattern among mosquito populations across the wide geographical range. First, genetic differentiation among *Ae*. *aegypti* populations were estimated through Wright’s F-statistic (*F*_*st*_) in FSTAT [50,51], and the statistical significance of the estimates was assessed by 10000 permutations. Heat maps using *F*_*st*_ values were constructed using complex heatmap package [52] in R v 4.0.3 [53]. Genetic clustering was observed using principal coordinate analysis (PCoA) in GenAlEx 6.5 for both individual samples and population-wise data. This was further complemented by the hierarchical clustering on principal components (*HCPC*) analysis with *FactoMineR* v.1.41 [54]. Clustering was also demonstrated through neighbor-joining tree based on genetic distance (corrected dst) using POPTREE [55]. The reliability of the tree topology was assessed by 1000 bootstraps. Finally, Bayesian clustering was performed in STRUCTURE 2.3 assuming admixture with correlated allele frequencies [56]. To determine the number of genetic clusters in the dataset a range of K values was tested from K = 1 to K = 10. Each K was simulated with Monte Carlo (MCMC) simulations using 200000 iterations and 600000 burn-in periods. The data were run using prior information of the population (LOCPRIOR) as well as without LOCPRIOR using similar parameters as mentioned above. The best possible K for both models was determined using Evanno *et al*., [57] ΔK method available in Structure Harvester [58]. The final bar plot of genetic ancestries determined by STRUCTURE software was visualized using the CLUMPAK server [59]. Finally, the partitioning of genetic variations was determined using AMOVA in GenAlEx 6.5.

### Role of geographic and climatic variables

The impact of geographic distance on the genetic structure between the populations was determined by Isolation-by Distance (IBD). As per the IBD hypothesis, the genetic distance between populations increases with the geographic distance between them. To test this, the Mantel test was performed between pair-wise genetic distance (*F*_*st*_/(1-*F*_*st*_)) and geographical distance using GenAlEx 6.5. To determine the impact at a finer scale; spatial autocorrelation analysis was done following Smouse & Peakall [45] as implemented in GenAlEx 6.5.

Finally, to determine the impact of climatic and geographic factors on the genetic structuring of *Ae*. *aegypti*, redundancy analysis (RDA) was performed using the vegan package [60,61] in R. RDA is a multivariate linear regression analysis that uses dependent and independent variables matrices for analysis. The membership coefficients for each cluster obtained from STRUCTURE were used as the dependent matrix and the geographic and climatic variables were used as two separate explanatory matrices. The 19 bioclimatic variables included were extracted from the WorldClim database, and geographic variables included; longitude, latitude, altitude, area, and population. Initially, Pearson’s correlation analysis was performed to quantify the relation between different variables. Among the variables that were highly correlated (R^2^>0.7), the more biologically meaningful variables were selected for further analysis which resulted in the selection of four climatic variables (temperature, temperature seasonality, precipitation, and precipitation seasonality) and three geographic variables (longitude, latitude, and altitude). To disentangle the impact of each type of variable three models were tested in RDA; (i) a full RDA model testing all variables (both climatic and geographical), (ii) a partial model in which geography explains genetic data keeping climatic variables as constant; and (iii) the third model determined the impact of geographical variables conditioned on climatic variables. This analysis determined the contribution of individual climatic and geographic variables, as well as the impact of the combination.

## Results

### Dataset analyzed

A total of 625 mosquitoes from 22 populations (20–32 mosquitoes per population) were analyzed using 12 microsatellite loci. None of the locus pairs were found in linkage disequilibrium after the Bonferroni correction (S2 Table). However, 40 out of 217 locus-specific tests deviated significantly from HWE (S3 Table). This might be due to the presence of null alleles as null alleles were identified in all the markers (S4 Table). Two microsatellite markers (AG5 and AC5) having null alleles in ≥50% of the populations were removed from the analysis. Null allele frequency in the remaining 10 markers varied from 0.05 in AC4 to 0.33 in CT2 (S4 Table). To see the impact of null alleles on genetic estimations, *F*_*st*_ analysis using FreeNA was determined. Overall *F*_*st*_ with and without correction was 0.088 and 0.094, respectively, (S5 Table) and the locus-wise *F*_*st*_ values were not significantly different from each other (t-test, = 1.061, p = 0.302). Moreover, the relationship between the null allele frequencies for each locus with the expected heterozygosity of each marker was not significant (p> 0.05). This signifies that the null alleles have a negligible impact on genetic estimations. Therefore, 10 loci out of 12 were retained for further analysis.

In addition to this, thirty-six individual samples having >30% missing alleles were removed from the data. The remaining 589 individuals showed 585 multilocus genotypes (MLGs); 581 of which were unique, while four MLGs were shared each by two mosquitoes. However, each mosquito belonged to a different population. Thus, the final dataset analyzed in this study included 589 individual mosquitoes genotyped using 10 microsatellite loci (A9, AC4, AC2, AG2, A1, B2, B3, AG2, AC5, CT2).

### Genetic diversity

A total of 139 alleles were identified among 589 mosquitoes from 22 locations. The number of alleles varied from 9 in AC4 to 30 in AG2 (S5 Table). All the microsatellite markers were polymorphic except three loci (AC4, B2, and AC1) which were monomorphic in WB1 and one (AC4) in AS1. The basic summary statistics of each marker as well as each population are given in S5 Table and Table 1, respectively. The mean number of alleles (*Na*) varied from 2.3 in WB1 to 6.0 in KR1 and an effective number of alleles (*Ne*) ranged from 1.8 in WB1 to 2.8 in TN2 (Table 1). In each population, *Ne* was lower than *Na*. This is because two to three high-frequency alleles observed in each locus were commonly observed in all the populations. While 78% of the alleles were shared by different populations, 30 alleles (22%) were private (found in a single population only). A maximum number (20%) of the private alleles were found in marker CT2 followed by 13% each in AG2 and AC1.

Overall, observed heterozygosity (*H*_*o*_) = 0.553 was similar to the expected heterozygosity *H*_*e*_ = 0.527 among all the 22 *Ae*. *aegypti* populations. However, the diversity level was variable among the populations. The Andaman & Nicobar Island (AN1, AN2 & AN4) populations showed the highest genetic diversity and the WB1 population showed the lowest (Table 1). Observed heterozygosity (*H*_*o*_) among populations varied from 0.205 in WB1 to 0.856 in AN1 and *H*_*e*_ varied from 0.354 in WB1 to 0.598 in AN4 (Table 1). Expected heterozygosity (*H*_*e*_) was higher than *H*_*o*_ among ten populations with significantly positive *F*_*IS*_ indicating an excess of heterozygotes. Among the remaining 12 populations, four showed significantly negative *F*_*IS*_ (Table 1).

### Genetic differentiation and population clustering

**Fig 2:**
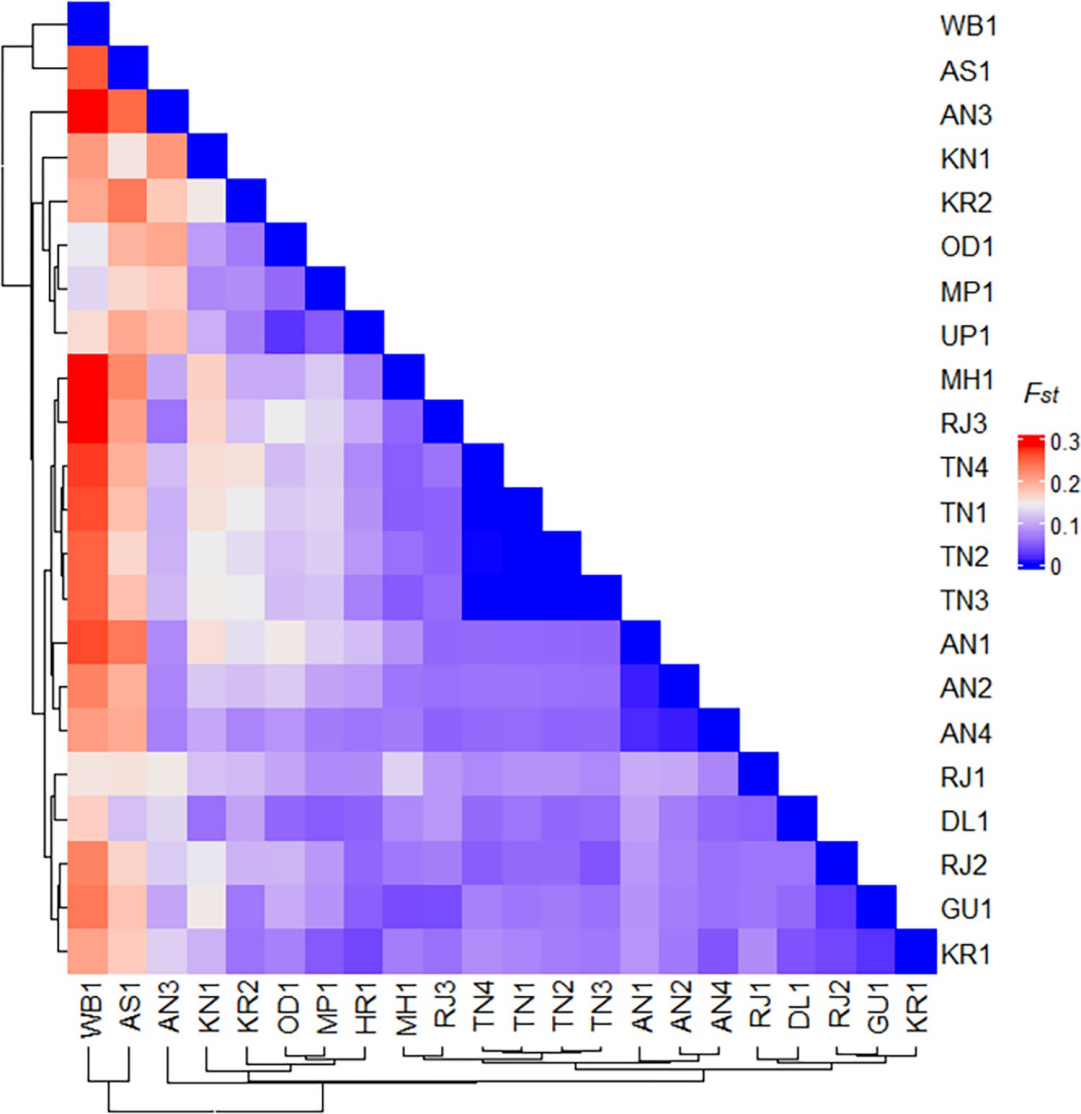
Heatmap of pairwise-*F*_*st*_ values of 22 *Aedes aegypti* populations from India genotyped using 10 microsatellite loci. Location codes are as given in Table 1.

A moderate level of genetic differentiation among 22 *Ae*. *aegypti* populations were observed with an overall *F*_*st*_ of 0.099 and gene flow (Nm) of 3.58. The *F*_*st*_ values varied from 0.00 (between TN populations) to 0.37 (between AN3 and WB1). Out of 231 pair-wise *F*_*st*_ comparisons, 80% were statistically significant (S6 Table). The remaining 20% values were non-significant due to very low *F*_*st*_ between some geographically closer populations such as the populations collected within TN and AN (Fig 2) indicating high genetic connectivity between them. AMOVA analysis demonstrated that 10% of the genetic variation was contributed by differences between populations, 2% within populations, and 88% were described by variations within individuals.

### Population genetic structure

**Fig 3:**
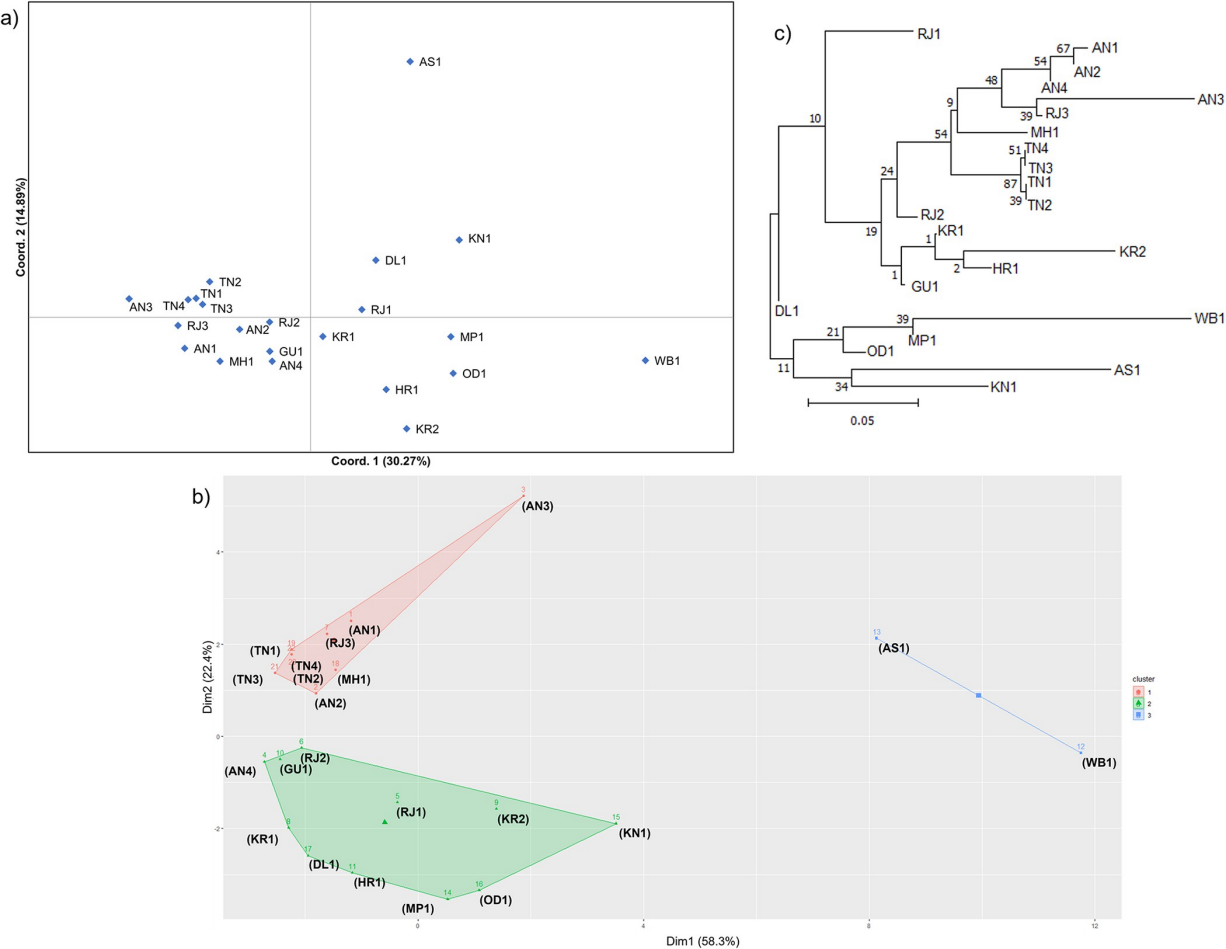
Genetic clustering of 22 *Aedes aegypti* populations from India based on microsatellites data, A) Principal Coordinate Analysis (PCoA) based on genetic distance between populations, B) Hierarchical Clustering on Principal Components (HCPC) plot for population-wise genetic data, C) Neighbor-joining tree based on corrected genetic distance (dst) between 22 populations constructed using POPTREE. Location codes are as given in Table 1.

Exploratory PCoA analysis of individual microsatellite genotypes revealed a large overlap among the samples from all the populations. However, some of the samples were differentiated (S2A Fig). The differentiation was also visible in population-wise PCoA (Fig 3A). The HCPC analysis grouped all 589 individuals into three clusters (S2B Fig) with overlaps between them. Similarly, HCPC divided 22 populations into three clusters, where GU1, RJ3, MH1, AN, and TN populations are in cluster 1 and WB1 and AS1 in cluster 3, and all other populations grouped in cluster 2 (Fig 3B). A similar pattern of clustering was observed through POPTREE (Fig 3C). Within cluster 1, populations from AN, TN, RJ3, and MH1 were found, and cluster 2 comprised of WB1, OD1, MP1, AS1, and KN1. The remaining populations were found separated from both clusters.

**Fig 4:**
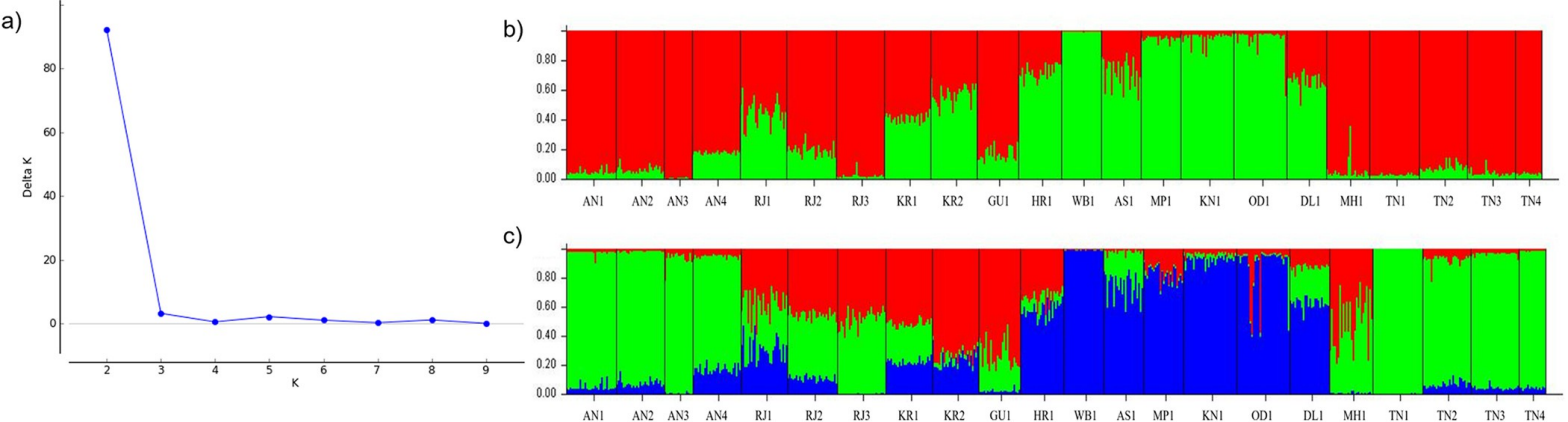
STRUCTURE analysis A) Delta K values for different number of populations assumed (K) in the structure analysis. Results of individual assignment tests for the 22 *Aedes aegypti* populations from India based on STRUCTURE analysis with LOCPRIOR model assuming B) K = 2 and C) K = 3. Each vertical bar represents one individual and the colors depict the genetic ancestry.

STRUCTURE analysis identified K = 2 as the best K value (Fig 4A). Bar plots for K = 2 and K = 3 are shown in Fig 4B and 4C, respectively. However, clustering among 22 *Ae*. *aegypti* populations were best described by K = 3 similar to the pattern observed by other methods as mentioned above. Based on the membership coefficient (Q>0.75), we grouped the populations into clusters. The populations from TN and AN grouped together forming cluster 1. The second cluster consisted of populations WB1, OD1, AS1, MP1, and KN1. The remaining populations had shared ancestries from both or either of the two clusters showing admixture. Notably, populations collected from within states (like TN, AN, RJ, and KR) were found genetically closer to each other (Fig 4C). However, populations from Delhi (DL1), Rajasthan (RJ) and Thiruvananthapuram (KR1) showed all the three ancestries in more than 10% of the total samples.

### Effect of geographical and climatic factors on genetic structure

**Fig 5:**
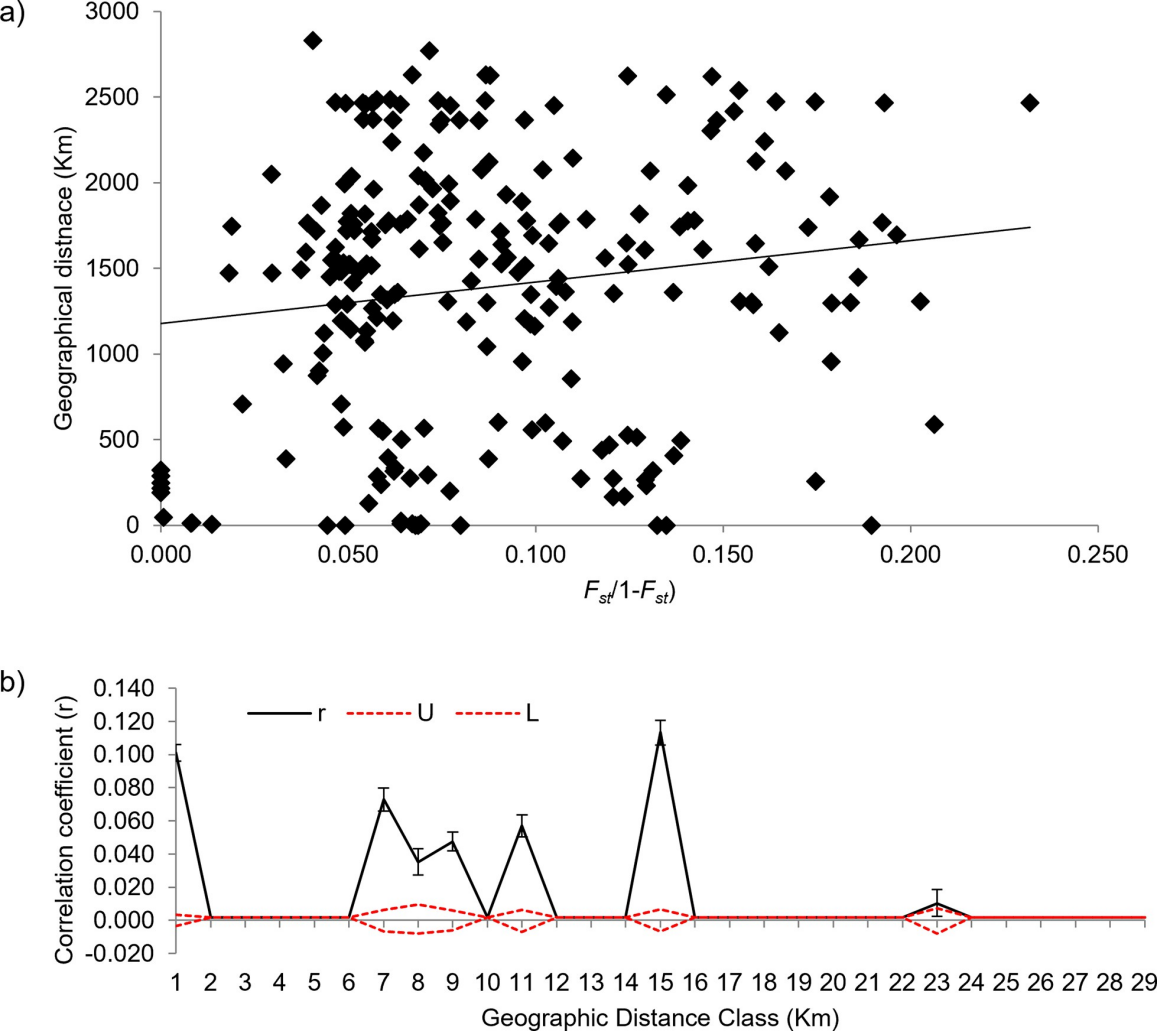
Isolation-by-distance (IBD) A) Mantel test between pair-wise genetic distance (*F*_*st*_/(1-*F*_*st*_)) and geographical distance between 22 *Aedes aegypti* populations from India, B) Correlogram plots obtained from spatial autocorrelation analysis for all the 22 *Aedes aegypti* populations. The autocorrelation index (r) is shown as a solid black line. Upper (U) and lower (L) values of 95% confidence interval are shown as two dotted lines. A 95% confidence interval determined by bootstrapping is shown as an error bar on the r graph.

The genetic differentiation between *Ae*. *aegypti* populations showed a clear signature of IBD (Fig 5A). A significant positive correlation (R^2^ = 0.023, p = 0.02) between the pair-wise matrixes of genetic differentiation (*F*_*st*_/1-*F*_*st*_) and geographical distances was observed. Spatial autocorrelation analysis indicated a significantly positive autocorrelation coefficient (r) only up to ~100 km. The genetic similarity diminished after ~100km, however, random peaks were observed at larger geographical distances (Fig 5B).

**Fig 6:**
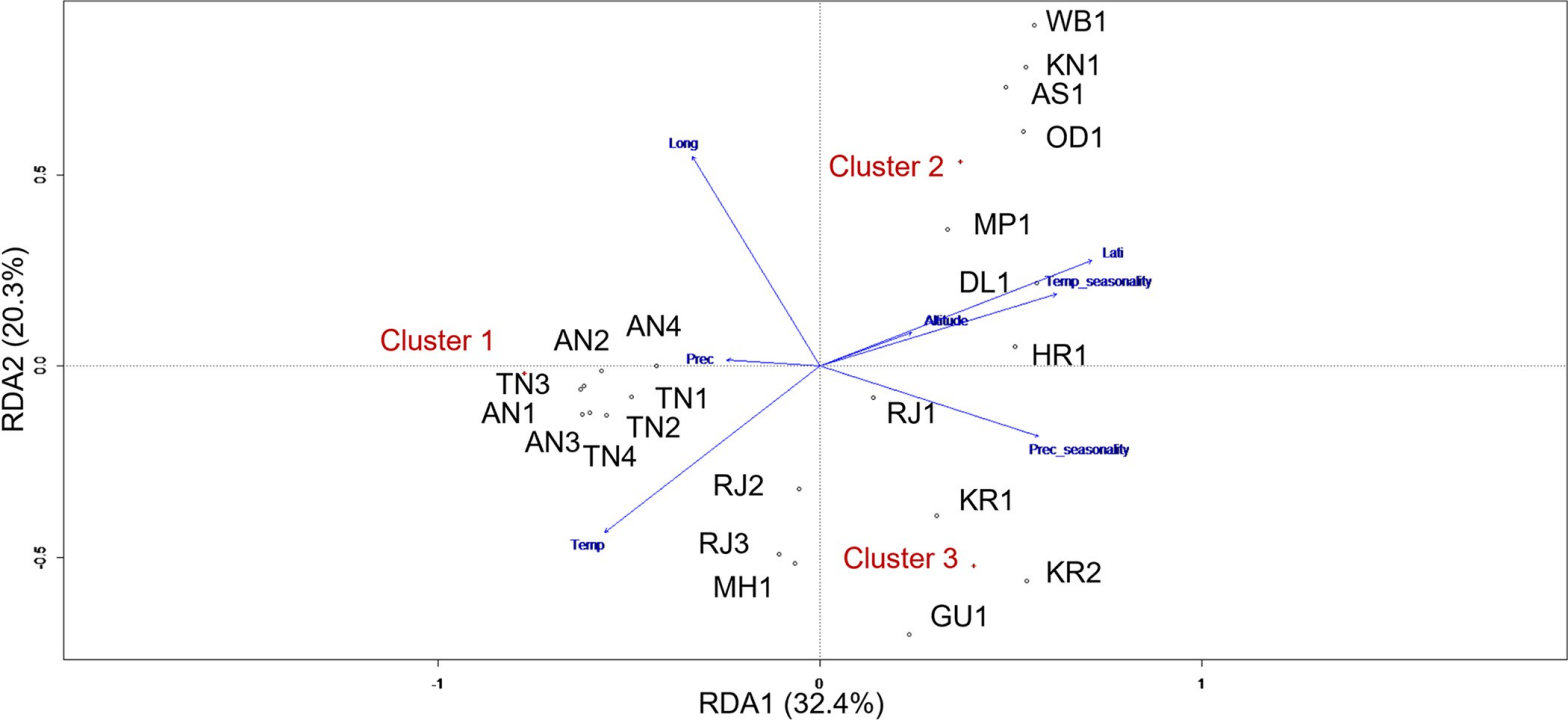
Full redundancy analysis plot showing the impact of climatic and geographic variables on genetic structuring of 22 *Aedes aegypti* populations from India.

Further, the impact of geographical and ecological factors on the genetic structuring of *Ae*. *aegypti* populations were determined using RDA analysis. The first two axes of the full redundancy analysis, which included geographic and environmental variables, explained 52.7% of the total genetic variation (Fig 6) with climatic factors accounting for 17.23% of the variation. Geographical factors explained 22.7% of the variation and the interaction between climate and space explained 29.11% (Table 2). Among all the variables, temperature, precipitation seasonality, longitude and latitude showed strong impact (Fig 6). However, only latitude and temperature were found statistically significant (p<0.05) among geographical and climatic variables, respectively.

**Table 2:** Summary of Redundancy analysis (RDA). * Indicate significant variables (p<0.05) based on 1000 permutations.

Variables		Variation explained	
Precipitation	Climatic variables	17.23%	-0.022
Precipitation seasonality			0.99
Temperature*			10.7
Temperature seasonality			13.97
Altitude	Geographical variables	22.7%*	-0.021
Longitude			0.073
Latitude*			16.21

## Discussion

This study represents the first large-scale genetic analysis of 22 *Ae*. *aegypti* populations from different geo-climatic zones of India. The study was conducted to determine whether *Ae*. *aegypti* populations across the country belong to a homogenous gene pool or are a mixture of con-specifics (locally adapted and genetically different) and how the genetic structure among populations is influenced by the local ecological and geographical factors. The genotyping of 589 mosquitoes with 10 microsatellite markers revealed high genetic diversity with moderate genetic differentiation among *Ae*. *aegypti* populations.

### High genetic diversity indicates continuous invasion and expansion of mosquito populations

High genetic diversity was observed among 22 *Ae*. *aegypti* populations from India. A total of 139 alleles were observed with an average of >4 alleles from each population. Among 589 mosquitoes, 585 unique multilocus genotypes were observed. Moreover, overall *H*_*e*_ was 0.527 and *H*_*o*_ = 0.553 which depicts a high level of genetic diversity as mentioned in Takezaki 1996 [62]. Observed heterozygosity (*H*_*o*_) in this study was similar to that observed among invasive populations from other countries (overall *H*_*o*_ = 0.529), however, less than the ancestral populations from Africa (*H*_*o*_ = 0.613) as determined by analyzing 79 *Ae*. *aegypti* populations worldwide [23]. *Aedes aegypti* is an invasive species that has existed in India even before the 19^th^ century [28,30,63]. The high level of genetic diversity might be due to its long-term establishment and exponential growth which is also supported by the negative *F*_*IS*_ value (-0.025). The high genetic diversity could also result from the continuous admixture of mosquito populations from other countries or geographical regions. *Aedes aegypti* is known to be transported as eggs or immature stages across countries [64] and due to extensive global connectivity, the chances of transportation have increased. Existing high-speed metro rails, extensive connectivity through airways and roadways, increasing trade across cities, migrations from rural to urban areas, excessive population growth and urbanization has facilitated the breeding as well as dispersal of mosquitoes across the country.

Expected and observed heterozygosity are important parameters in genetic diversity analysis. The difference between the two is determined by *F*_*IS*_ and the significance of *F*_*IS*_ indicates the complexity of genetic structure. Although overall *H*_*e*_ and *H*_*o*_ were almost similar, the level of diversity varied across the populations. Around half (10/22) of the populations had *H*_*e*_>*H*_*o*_ with significant positive *F*_*IS*_ indicating a deficit of heterozygotes which happens due to inbreeding among relatives [65]. The highest *F*_*IS*_ was observed in WB1 followed by AS1, KN1, DL1, and others. Most of these populations belong to big cities/municipalities or the state capitals where mosquito control is more active compared to smaller cities/towns. Regular vector control could cause a reduction in mosquito effective population size through bottleneck and genetic drift leading to a loss of diversity as seen in the vector populations from Trinidad and Tobago, and Venezuela [66–68]. Indeed, loss of heterozygosity may also result from sampling errors. For example, sampling the related individuals from a single location or genotyping multiple samples from one larval container may not represent the genetic diversity of the study locations. In this study, the majority of the samples were collected through larval/pupal collections and we have considered only one adult from each container to avoid analyzing siblings. *Aedes* mosquitoes having low dispersal capabilities (<500m) [13–17] can lay eggs in several containers lying nearby [69,70]. However, to overcome this we collected samples from more than one location (>500m apart) within each study site and genotyped 20 to 32 mosquitoes from each population which is generally considered sufficient to represent the genetic diversity of the sampling site [71].

### Complex genetic structure

Clustering analysis based on PCoA, HCPC, POPTREE, and Bayesian approach consistently identified two major genetic clusters. One cluster consists of populations from Tamil Nadu (TN) and Andaman & Nicobar (AN), and the other from eastern India (Kolkata, Odisha, and Assam), Madhya Pradesh and Bangalore. The remaining nine populations *i*.*e*., 41% of the total populations spread across India showed an admixture of genetic ancestries. Based on the membership coefficient of the identified genetic ancestries through STRUCTURE, three populations (MH1, GU1 and RJ3) shared ancestry with cluster 1, two population (HR1 and KR2) shared with cluster 2 and the remaining four populations (DL1, RJ1, RJ2, KR1) had all the three identified genetic ancestries. This type of genetic structure is referred to as “chaotic genetic patchiness” [72]. The populations that are geographically closer (TN, KN1, KR) are genetically structured but chaotic relatedness among widely located populations (*e*.*g*., KN and AS, RJ and TN) was observed. This indicates that apart from the geographic distance, other factors determine the genetic relatedness between the mosquito populations. One of the widely known factors is the human-mediated dispersal of mosquito eggs, larvae and adults across broader geographical ranges [73]. Several studies have reported high levels of genetic differentiation within the urban areas but low differentiation between the urban areas [18,19,74–76]. Due to low dispersal abilities of the mosquito species, this is possible through the passive dispersal. Majority of the populations in this study are from commercially important urban centers in India which are well connected and have significant movement and trade connections with other geographical regions across the country. For example, Delhi (DL1) is the capital of the country, Rajasthan (RJ) and Kerala (KR) are the major touristic states in north and south of India. The mosquito populations from these states showed mixture of all the three genetic ancestries. Thus, connectivity networks and the rate of transportation are the important parameters in determining the mosquito genetic structure.

In this study, there are two major observations from clustering analysis. One is the sharing of genetic ancestries across a broad geographic range indicating the admixture. The possibility of long-distance dispersal was also reflected in the IBD analysis. Although mantel test showed a significant positive relation (R^2^ = 0.023, p = 0.02), between geographic and genetic distance, the r^2^ was very low indicating a weak relation possibly due to the existence of long-distance gene flow. This is further supported by the spatial autocorrelation analysis which demonstrated the absence of genetic connectedness beyond ~100km, however, several random peaks were observed for higher geographical distances. Notably, the populations collected within states/UT (such as Tamil Nadu, Port Blair, and Rajasthan) were found genetically closer. This might be due to connectivity which is more frequent within states compared to between states and thus could be responsible for population admixtures.

The second important observation of the clustering analysis is the identification of two genetic clusters with *F*_*st*_ = 0.11, p = 0.02 between them. The genetic differentiation might be influenced by ecological as well as geographical factors as in cluster 2 the populations belonged to the eastern region except for KN1, and MP1 and cluster 1 contained four populations each from TN and AN. TN and AN have a major geographical barrier (Ocean) between them, however, our results indicate that either there is a continuous exchange of mosquito populations between these areas or the populations once introduced have not gone through enough generations to develop significant differences between them.

Furthermore, the existence of genetic clusters among *Ae*. *aegypti* populations can be explained by two hypotheses. One hypothesis is that the mosquito populations in each cluster might have entered separately from other countries or geographical regions [77,78]. Each cluster has some geographical and ecological variations and has varied levels of connectivity with the neighboring countries. For example, the Eastern parts of the country are closer to Thailand, China, Bangladesh, and Myanmar while the Southern part is more connected to Sri Lanka and Maldives. The genetic background of *Ae*. *aegypti* populations in different countries differ from each other [23]. Nonetheless, this hypothesis remains to be tested by comparing our samples with those from other countries. The second possibility of this type of structuring among *Ae*. *aegypti* populations in India is that the mosquito species is strongly associated with humans and their surroundings and do not fly to far-off places if enough breeding places are available nearby [13–17]. Thus, its low dispersal capabilities and local adaptation due to anthropological as well as environmental factors might be developing genetic differentiation over time [79–83]. However, further sampling of populations from these two clusters and the areas between them seems important for a full understanding of the contemporary structure of *Ae*. *aegypti* populations and the factors responsible for that. Moreover, an in-depth investigation of these two clusters would be interesting to understand if there is any difference in the level of vector competence, insecticide resistance, or other features related to the transmission of vector-borne diseases.

### Impact of environmental factors

*Aedes aegypti* is closely associated with human dwellings, and thus its genetic structure is known to be influenced by anthropological and environmental factors like availability of breeding places, the intensity of vector control activities, connectivity, human population density, land use pattern, rainfall, temperature, etc. [1–3,84–86]. In this study, we elucidated the impact of some of these factors to determine whether the observed genetic structure was caused by geographical distance, climatic conditions, geographical conformation, or a combination of climatic and geographic factors. Our IBD analysis revealed a positive relationship between genetic and geographical distance indicating that geographical isolation could be one of the causes of the genetic diversity of *Ae*. *aegypti* in India. However, weak relations and the observations from autocorrelation analysis indicated the chances of long-distance gene flow between the populations indicating the role of other factors in shaping the genetic structure.

RDA analysis identified that 52.7% of the genetic variation in the mosquito species is explained by the selected climatic and geographical variables. Among all the variables, latitude, and temperature contributed significantly. Geographical factors were found to be more important than climatic factors indicating that mosquito genetic structure is influenced by the factors associated with geographical locations. Notably, around half of the variation remained unexplained. There are many other important factors like number of breeding places, land use pattern, population size, infrastructure, vegetation, etc. that have not been analyzed in this study due to unavailability of the data estimates.

## Conclusions

Overall high genetic diversity in *Ae*. *aegypti* was observed, probably due to its long-term establishment and continuous expansion across the country. Three genetic clusters were identified. Two clusters were moderately differentiated from each other. The third cluster contained populations with shared genetic ancestries reflecting genetic connectivity among populations irrespective of geographical locations or distance between them. Although signs of genetic connectivity at a larger geographical distance were observed the species seems to be structured largely due to the impact of local factors associated with geographic locations. The populations within state/union territories were genetically similar indicating the role of human movements and connectivity on mosquito dispersal which is more frequent within the states than between the states. Vector management has to be focused at the state level considering the means of connectivity and the environmental factors which can dilute the impact due to gene flow from other areas. Further, fine-scale analysis of *Ae*. *aegypti* populations at the state level could be insightful to determine the role of possible factors on the genetic structure and dispersal of this mosquito species in understanding dispersal patterns of mosquitoes and thus guiding the effective vector management.

The results of this study improved the overall understanding of *Ae*. *aegypti* population dynamics across India and also facilitated the development of a large-scale reference base for future genetic studies in the country. Moreover, the data would provide a basis to predict the mosquito dispersal patterns across geographical regions, which is important for formulating effective control strategies as well as assessing the risk of disease transmission. Understanding genetic connectivity across climatic zones is particularly important in India which encompasses huge climatic variations from extreme cold in the northern parts to the tropical climates in the southern regions. The rainy season in India is generally July to September. However, the southern coast that includes the parts of Tamil Nadu receives around half of its annual precipitation from the retreating monsoons extending the rainy season from October to December [87,88]. This means if mosquitoes have means to travel from Tamil Nadu to other parts of the country, the mosquito season which is significantly associated with rainy seasons [89–91] may get prolonged in those areas and can also increase the chances of spreading the diseases. In depth entomological investigations are needed to determine the actual movement of mosquitoes through transportation facilities across geographical regions, frequency, migration route and the factors supporting it. This could guide the national vector control program for effective vector management and can also be related with the epidemiology of mosquito-borne diseases.

## Supporting information

S1 Fig A two-dimensional PCA plot obtained from a multiple factor analysis (MFA) performed on all 22 populations using 142 bioclimatic variables retrieved from the WorldClim database. (XLSX)

S2 Fig Genetic clustering of 22 *Aedes aegypti* populations from India based on microsatellites data. A) Principal Coordinate Analysis (PCoA) based on genetic distance between each mosquito sample, B) Hierarchical Clustering on Principal Components (HCPC) plot for individual mosquito samples.
(XLSX)

S1 Table Nineteen bioclimatic variables retrieved from WorldClim database using principal coordinates for each sampling site. (XLSX)

S2 Table The summary of linkage disequilibrium between each marker pair. (XLSX)

S3 Table Hardy Weinberg test per locus and per sample locations. Sample location codes are given in Table 1. The populations showing significant values after Bonferroni correction (p<0.0002) shown as *.
(XLSX)

S4 Table Frequency of null alleles identified in each microsatellite marker among all the 22 *Ae*. *aegypti* populations. (XLSX)

S5 Table The summary statistics of 10 microsatellite markers used to genotype *Ae*. *aegypti* populations in India. (XLSX)

S6 Table Population Pairwise *F*_*st*_ among 22 populations of *Ae*. *aegypti* in India. Codes for the population names are given in Table 1. Significant *F*_*st*_ values after Bonferroni correction (p<0.00024) are indicated in bold.
(XLSX)

S1 Data Microsatellite genotyping data for 22 *Aedes aegypti* populations collected from India. (XLSX)
